# The effect of media aids in genetic carrier screening education among patients with infertility

**DOI:** 10.1016/j.xfre.2025.01.003

**Published:** 2025-01-09

**Authors:** Annabelle Gordon, Anthony Leonard, Suruchi Thakore, Kurt Peterson, Emily Hurley, Megan Sax

**Affiliations:** a Department of Obstetrics and Gynecology, University of Cincinnati Medical Center, Cincinnati, Ohio; b Department of Family and Community Medicine, University of Cincinnati Medical Center, Cincinnati, Ohio; c IVF Michigan and Ohio Fertility Centers, East Lansing, Michigan; d Department of Reproductive Endocrinology and Infertility, University of Cincinnati Medical Center, Cincinnati, Ohio; e Advanced Fertility Care, Phoenix, Arizona

**Keywords:** Genetic carrier screening, EngagedMD, multimedia education

## Abstract

**Objective:**

To evaluate the effect of educational videos on the utilization of preconception genetic carrier screening in patients with infertility.

**Design:**

Survey study.

**Subjects:**

New patients presenting for infertility consult with one of five providers (4 physicians and 1 nurse practitioner) from November 2021 through February 2022.

**Exposure:**

Patients were assigned to the web application EngagedMD video arm or in-person counseling arm for education on preconception genetic screening.

**Main Outcome Measures:**

The primary study outcome was completion of genetic carrier screening. The secondary outcomes were provider rating of patient comprehension and provider-rated time demand of counseling.

**Results:**

A total of 73 patients were enrolled: 42 in the video arm and 31 in the in-person counseling arm. The survey response rate was 100% for patients and providers. Patients assigned to the video counseling arm were significantly more likely to plan to proceed with carrier screening (78.6%) than those receiving only in-office counseling (41.9%). The odds ratio associating video arm with actual completion of screening was 5.07 (95% confidence interval, 1.84–13.96). Patients who completed EngagedMD videos were perceived by providers to have a noninferior understanding of the purpose of carrier screening compared with those who underwent standard counseling, assessed via “teach-back” method. Providers also rated that multimedia education use significantly decreased demand on appointment time.

**Conclusion:**

The use of patient education videos increases utilization of preconception genetic carrier screening and is an acceptable alternative to in-person provider counseling.

Genetic carrier screening is the testing for underlying allele variants that are associated with genetic disorders in individuals who do not outwardly display any disease characteristics (1). A patient undergoes genetic carrier screening for the purpose of identifying his or her specific risk of having a child with a disorder of autosomal recessive inheritance.

Offering genetic carrier screening to all patients who are pregnant or considering pregnancy, regardless of race or ethnicity, has been recommended by the American College of Obstetricians and Gynecologists (ACOG) since 2011 (1). The traditional method for carrier testing was ethnic-based screening or targeted screening for disorders of higher prevalence in the patient’s specific ethnic group (2). However, as our society becomes increasingly diverse and racially mixed, panethnic screening and expanded carrier screening have become more commonplace. Panethnic, or nondirective, screening involves testing a patient for genetic disorders that were historically only tested in specific ethnic groups, regardless of the patient’s ethnicity (2). This typically involves screening for a handful of genetic disorders. In contrast, expanded carrier screening tests for >100 different genetic disorders of varying degrees of clinical prevalence and is made possible by the rapid advances in DNA sequencing in recent years (2). Although ACOG does not distinguish one method as superior, it has continued to expand and reaffirm its recommendation that all women be offered some form of carrier screening (2).

Although these recommendations have existed for over a decade, the decision to proceed with carrier screening is far from universal. For example, Ekstrand Ragnar et al. (3) found that just one third of couples were interested in undergoing preconception carrier screening. Another study demonstrated that up to 24% of patients will have positive screening for at least one genetic condition on expanded carrier screening, although only an estimated 5% of patients use expanded carrier screening in the United States, although this number is not formally tracked (4). In a later study, Lazarin et al. (5) again estimated that only 5% of pregnant patients in the United States underwent expanded carrier screening in 2015, 4 years after ACOG’s initial recommendation for universal preconception screening. There are a multitude of reasons why patients do not more commonly undergo carrier screening of any variety, although past studies have found that limited clinician experience with screening modalities, limited clinician time, and patient confusion surrounding the subject are pervasive issues (6, 7). The scarcity of genetic counselors in the United States, with only one per 82,000 people, is likely an additional contributing factor (7).

Because of advancements in reproductive health, there are interventions available to those with positive carrier screening, such as donor gametes or in vitro fertilization (IVF) with preimplantation genetic testing, as well as other options such as early prenatal screening/diagnostic testing or adoption. Because positive results are common and can be difficult to navigate, it is important for patients to make informed decisions concerning whether to pursue genetic carrier screening. Because expanded carrier screening options can identify variants of unknown significance, provoking significant anxiety and perhaps prompting unnecessary medical interventions, this topic can be even more fraught. Provider counseling regarding these options is an imperative part of preconception or early prenatal care.

One way the medical field has sought to improve patient counseling in recent years is through the addition of multimedia education (MME) platforms. These online platforms employ a combination of audiovisual tools and comprehension questions to improve patient education. The goal of MME implementation is to provide a range of benefits to both patients and providers. These can include saved time during appointments as well as improved patient comprehension because the healthcare setting can cause anxiety for patients, leading to decreased information retention (8). Multiple recent studies have explored the application of these tools to a broad range of obstetrics and gynecology topics, although results have varied regarding the tools’ superiority to traditional patient education. Qureshey et al. (9) found a significant increase in long-acting reversible contraception usage in individuals with high-risk pregnancies who used MME compared with those who underwent only routine counseling. Tucker et al. (10) found improved patient satisfaction and no difference in patient comprehension with use of an MME followed by targeted physician counseling vs. standard physician education alone for those undergoing surgery for endometrial cancer. However, the study found that MME usage led to a significantly longer appointment time (10). Pandya et al. (11) found no significant difference in anxiety, knowledge, or satisfaction between patients counseled on uterine fibroids by a physician vs. an MME.

To our knowledge, no study has examined the utility of an MME for the topic of preconception genetic carrier screening in the trying to conceive population. Although a recent study by Conijn et al. (12) did evaluate genetic carrier screening knowledge in a reproductive age population after consumption of an educational video compared with an educational paragraph, this study did not examine trends in patients actively seeking pregnancy or in a clinical setting. Two other recent studies examined MME use related to genetic counseling in patients who were already pregnant, with one evaluating patients who had already had positive fetal screening for Down syndrome (13, 14). As ACOG recommends carrier screening before conception, our study has unique clinical implications. Furthermore, no study, to our knowledge, has examined MME usage compared with standardized physician counseling for this topic. However, two recent studies have demonstrated that the MME platform EngagedMD, an online application that offers patient education tools for a variety of healthcare fields, including reproductive endocrinology and infertility (REI), was associated with increased patient comprehension and improved informed consent for the assisted reproductive technology (ART) process (15, 16). We hypothesize that the usage of this standardized counseling tool will improve patient understanding of genetic carrier screening and that patients who complete counseling with this model will be more likely to undergo carrier screening before ART.

## Materials and methods

This prospective survey study was conducted at the University of Cincinnati (UC) Center for Reproductive Health. The study was approved by the UC Institutional Review Board. Data collection took place from November 2021 through February 2022 at the UC Center for Reproductive Health’s three clinic locations.

We partnered with EngagedMD, an MME platform, for this study. For patients to access educational tools via this platform, a medical office should have an agreement in place with EngagedMD and assign the relevant content to their patients.

The study participants were new patients presenting to UC for their initial infertility consultation with one of five providers (4 REI physicians and 1 nurse practitioner). The exclusion criteria included those who were non-English speakers, those who had their initial consultation via telehealth, and those who had previously completed genetic carrier screening or were already known carriers. All eligible patients were contacted by telephone and consented to participate in this study before their new patient visit. Once consented for the study, patients were assigned to receive either the standard, in-person physician counseling regarding carrier testing (conventional group) or the MME videos and comprehension questions via the EngagedMD platform (intervention group). Patients were assigned to either the conventional counseling or intervention group on the basis of the month in which their initial counseling visit took place.

Those who were assigned to the intervention group were sent the link to the EngagedMD video library and asked to complete the set of videos before their initial appointments. These patients also received standard carrier screening counseling at the time of their appointments. EngagedMD provided five videos titled “Intro to Carrier Screening,” “How Diseases are Inherited,” “X-Linked Conditions,” “How Carrier Screening Works,” and “Options for High-Risk Results.” The videos totaled 12 minutes of material. The videos were available to be paused, rewound, and rewatched. Associated comprehension questions, called “knowledge checkpoints,” after each video were also provided as a part of the modules. We were able to verify via the EngagedMD platform whether patients completed the assigned videos.

Patients who were assigned to the standard physician counseling arm discussed the topic of carrier screening during their initial visit with the provider. Talking points were given to the providers to reference during each appointment to ensure that counseling was standardized among patients in both groups (Supplemental Fig. 1, available online).

Immediately after the infertility consultation in the office, patients of both groups were asked to “teach-back” the purpose of preconception carrier screening to the provider and to complete a paper survey about their experience and understanding of genetic carrier screening (Supplemental Figs. 2 and 3). The surveys for both the video arm and standard counseling arm each had four questions that were structured as a Likert scale. Both surveys also asked patients whether they planned to complete screening as a yes-or-no question. The survey for the video arm participants additionally asked if participants had completed the EngagedMD videos. Providers also completed a survey to assess patient’s understanding and time spent counseling (Supplemental Fig. 4). Providers were not blinded to the patient’s group.

The primary endpoint of the study was the decision to undergo genetic carrier screening by the patient. Patients reported whether they planned to undergo carrier screening, and completion of carrier screening was confirmed in the electronic medical record. The secondary endpoints were provider opinion of patient comprehension of genetic carrier screening and provider assessment of demand on physician time caused by carrier screening counseling. Provider opinion of patient understanding was evaluated via the “teach-back method” in which the providers asked patients to explain the purpose of carrier screening at the conclusion of the visit. Provider-rated patient comprehension was assessed on the basis of a patient’s ability to highlight the purpose of genetic carrier screening, common recessive inheritance disorders, different carrier screening panels, and reproductive options if both partners are found to be carriers for the same genetic condition and reported via a Likert scale (Supplemental Fig. 4). A form consisting of these points was given for providers to reference during each appointment (Supplemental Fig. 1).

The statistical analysis of survey results compared the intervention and control groups using the Fisher’s exact tests for comparing rates or distributions and Wilcoxon’s tests to compare levels of directional scales (e.g., age or responses of 1–5 for strongly agree to strongly disagree). The primary outcome of accepting genetic counseling was further examined using logistic regression to adjust for which of the three clinics was attended by the patient, patient age, and patient race, with each of those three factors included one at a time. Furthermore, race was examined using White and Black patients only, to estimate the interaction between race and treatment group in their effect on the primary outcome. The study α was a two-tailed *P* value of .05, and all analyses were conducted using Statistical Analysis System. The study was powered assuming a rate of planned screening of 30% in the control group and a two-tailed α of 0.05. By assuming a rate of 70% in the intervention group, an 80% power was achieved using 30 patients per group, whereas by assuming a rate of 60% in the intervention group, an 80% power was achieved using 50 patients per group. The study was continued until our sample sizes were within that range.

## Results

**Figure 1:**
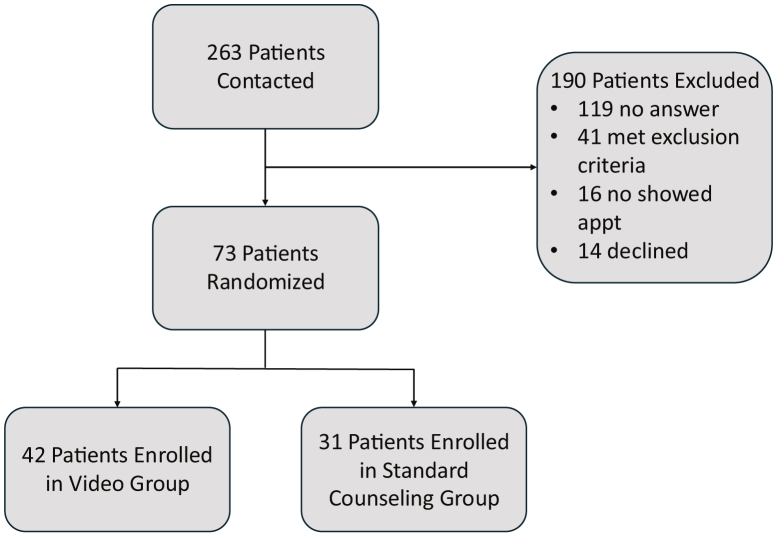
Patient enrollment diagram.

From November 2021 through February 2022, 263 patients were scheduled for an initial infertility visit and were recruited by telephone. Of this group, 119 patients did not answer or return our telephone calls, and 41 met the exclusion criteria. An additional 14 patients declined to participate in the study, and 16 patients enrolled in the study but did not ultimately present for their initial appointments. A total of 73 patients enrolled in the study and were included in the analyses, with 42 patients assigned to the intervention group and 31 assigned to the control group (Fig. 1). There were no significant demographic differences between the video and nonvideo groups (Table 1).

**Table 1:** Patient demographic information.

Demographic variable	Video	Nonvideo	*P* value
	N = 42 (58%)	N = 31 (42%)	
Age (mean [SD])	30.3 (5.9)	29.1 (4.7)	.50
Race (N [%])			.34
White	28 (67%)	25 (81%)	
Black	10 (24%)	6 (19%)	
Hispanic	1 (2%)	0 (0%)	
Other	3 (7%)	0 (0%)	

*Note:* “Age” is reported as the mean (SD), and “Race” is reported as the number (percentage). Groups were compared on age using a Wilcoxon’s test and on race distribution using the Fisher’s exact test.

The survey response rate was 100% for both patients and providers. Four patients (9.5%) assigned to the video counseling arm did not complete all videos. Regarding the primary endpoint of completion of genetic screening, patients who completed the video counseling before their new patient infertility appointment were more likely to plan to undergo genetic carrier screening at the conclusion of their appointment (78.6%) compared with those who received only in-office standard physician counseling (41.9%) (*P*=.003) (Table 2).

**Table 2:** Results from patient and provider surveys.

Survey response	Video	Nonvideo	*P* value
Planned screen	33 (79%)	13 (42%)	.003
Provider survey: the patient demonstrated a “good understanding” of preconception carrier screening via “teach-back method.”	1.8 (1.1)	2.1 (0.9)	.06
Provider survey: the patient appeared comfortable with decision to pursue testing or sign the waiver.	1.7 (0.9)	1.9 (0.8)	.20
Provider survey: the counseling put unusual demand on physician time.	3.5 (0.9)	2.7 (0.8)	<.001
Patient survey: I understand the reason for recommending preconception genetic carrier screening.	1.3 (0.6)	1.3 (0.4)	.44
Patient survey: I am comfortable with my decision to either obtain preconception genetic carrier testing or sign the waiver.	1.2 (0.5)	1.4 (0.5)	.09
Patient survey: I am satisfied with the preconception genetic carrier screening education/counseling I received.	1.1 (0.3)	1.4 (0.6)	.009

*Note:* “Planned screen” is reported as the number (percentage) of patients planning to undergo screening. Survey items are reported as means (SD) from a Likert scale with 1 indicating “strongly agree” and 5 indicating “strongly disagree.” *P* values comparing groups were from the Fisher’s exact test for screening and Wilcoxon’s test for the questionnaire items. Patients not enrolled in the study due to exclusion criteria were not included in this table.

The unadjusted odds ratio (OR) associating the video group with planned screening was 5.08 (95% confidence interval, 1.82–14.16), and that associating the video group with actual completion of screening was 5.07 (95% confidence interval, 1.84–13.96). These ORs varied because four patients reported that they planned to complete carrier screening but ultimately did not do so and two patients who reported that they did not plan to complete carrier screening actually did. On chart review, insurance coverage was often cited as a barrier for actual completion of screening. The effects of clinic, age, or race on the outcome were not significant and impacted the intervention OR by less than approximately 10% (Table 3). For the race-specific analyses, we examined only White and Black patients because there were very few patients in other racial categories. The interaction between the intervention and race had a *P* value of .46, indicating no significant racial difference in the effect of the intervention on planned screening. Black patients had a nonsignificantly higher rate of planned screening than White patients, 75% to 50%.

**Table 3:** Results for planned completion of genetic carrier screening and actual completion of genetic carrier screening with adjustments for clinic location, patient age, and race.

Result adjustment	Planned screening		Actual screening	
	OR (95% CI)	*P* value	OR (95% CI)	*P* value
No adjustment	5.08 (1.82–14.16)	.002	5.07 (1.84–13.96)	.002
Adjusted for clinic location	5.68 (1.86–17.31)	.002	4.78 (1.66–13.77)	.004
Adjusted for patient age	5.06 (1.81–14.21)	.002	4.92 (1.77–13.66)	.002
Adjusted for race	4.38 (1.54–12.44)	.006	4.37 (1.55–12.36)	.005

*Note: P* values from the Wald tests from the logistic regressions. CI = confidence interval; OR = odds ratio.

The secondary endpoints, as well as other results, from the patient and provider surveys are presented in Table 2. Responses were provided via a Likert scale. Patients in the video intervention arm had significantly higher satisfaction with their genetic carrier screening education (average rating, 1.1 vs. 1.4; *P*=.009). Although these patients also had higher comfort with their decision of whether to undergo screening (1.2 vs. 1.4, *P*=.09) and were rated by their providers to have greater understanding of the concept of carrier screening (1.8 vs. 2.1, *P*=.06), these findings were not statistically significant, and absolute differences between group means were very small and, therefore, likely without clinical significance as well.

The study also found that using an MME put less demand on providers’ time. Providers rated that counseling patients in the intervention arm regarding carrier screening placed significantly less demand on their time than those in the control arm (3.5 vs. 2.7, *P*<.001). In fact, providers marked that counseling placed an increased demand on their time in over 35% of control group visits.

## Discussion

Our data suggest that patients who use an MME tool for genetic carrier screening before in-person counseling are more likely to undergo screening than those who do not use the MME. In addition, the use of an MME was associated with higher levels of patient satisfaction and a subjective decreased demand on physician time. These findings suggest that implementing an MME tool for genetic screening would be beneficial to both patients and providers.

The EngagedMD videos were able to be paused, rewound, and rewatched and had associated comprehension questions. These attributes have all been documented to improve patient comprehension. A systematic review by Lewis (17) found that tools that were able to be individually paced were associated with improved learning. Lewis et al. (17) also highlight having an interactive quality as being particularly useful. Using a variety of media, such as videos with infographics and comprehension questions, helps appeal to a variety of learning styles, improving comprehension (8). Furthermore, healthcare settings are known to be anxiety-provoking, a state that is associated with poor comprehension and memory (8). This is avoided by allowing patients to watch the videos at home at their convenience. Additionally, the videos avoid unexplained medical jargon, which is known to be ubiquitous in provider-patient conversations and associated with poor patient comprehension (18).

As the United States only has one genetic counselor per 82,000 people, the topic of genetic carrier screening is typically broached and further explained by obstetricians and gynecologists (7). The time required for adequate explanation of this matter can be significant, considering its scientific complexity as well as the emotional burden. An additional challenge of these discussions is that there is no “right answer”; although universal preconception carrier screening is recommended, no single panel is favored, leading to confusion among patient (2). Our study demonstrated no significant difference in provider-rated patient comprehension between the video and standard counseling groups. Given this finding, providers can feel empowered to use an MME, such as EngagedMD, to offset some of this discussion, allowing appointment time to be directed toward patients’ specific questions and concerns regarding this topic.

Furthermore, our study found that MME utilization was also associated with a decreased subjective burden of demand on physician time. Physicians are faced with an ever-increasing demand to see more patients in less time. A recent study demonstrated that physicians experienced time pressure in 67% of new patient visits in primary care (19). This pressure is often felt during initial infertility evaluations when a full history, often of two people, should be obtained in addition to a significant discussion of workup and management options. Furthermore, patients can have significant anxiety or distress during this appointment, requiring the provider to adeptly manage both the medical and emotional contexts of the appointment in a timely fashion. The implementation of an MME in our study was associated with a significant subjective decrease in time burden on the physician, which allows for time to be redirected to other aspects of the visit and patient counseling. Although this study did not measure the actual differences in either total appointment time or time discussing carrier screening between the two groups, this could be an interesting area of further study.

Racial and ethnic disparities are widespread in healthcare, and the field of REI is no different. Racial minorities use ART services less than their White counterparts and have poorer ART and IVF outcomes (20). Genetic testing is another area of medicine in which racial disparities have been noted. A study by McQueen et al. (21) found that Black, LatinX, and low–socioeconomic status patients with infertility were significantly less likely to undergo genetic carrier screening than their White, higher-income counterparts. A benefit of using an MME is that it standardizes the counseling a patient receives, controlling for the implicit bias of the counselor. Many studies have demonstrated how implicit bias negatively impacts patient encounters (22, 23). This is particularly important as Lowe et al. (24) has demonstrated that genetic counselors with higher levels of implicit bias were less likely to offer individualized counseling to their minority patients. This phenomenon could be partially responsible for the smaller numbers of minority patients nationally who opt to undergo carrier screening. Our study found that the association between the intervention and intended screening rates was higher in White patients than in Black patients, although not significantly. However, this represents an area for future study.

To our knowledge, our study is the first to examine the implementation of an MME for a specific aspect of preconception counseling in the trying to conceive population as well as the association between MME usage and actual completion of preconception genetic carrier screening. The EngagedMD platform examined in this study was found to be statistically significantly associated with a higher likelihood of undergoing genetic carrier screening, as well as increased patient satisfaction and decreased subjective demand on physician time.

Additionally, in our clinic, patients would frequently defer carrier screening until the conclusion of their infertility workup, when they would be otherwise ready to initiate an IVF cycle. This can result in a delay of cycle start with any positive result. With increased screening rates after MME utilization, this delay could be avoided.

The limitations of this study include the fact that standard physician counseling varies between providers. In addition, patients undergoing an infertility workup are a population known to undergo carrier screening at higher levels, limiting the generalizability of our study to patients of generalist obstetrics providers (25). Furthermore, the smaller sample size likely limited our ability to adjust for multiple confounders simultaneously as well our ability to further elucidate differences in the mean survey responses between groups. Additionally, because our study population was small, imbalanced groups (42 in the video arm and 31 in the standard counseling arm) could have biased results. Lastly, this study was not blinded, and physicians were aware that patients had watched the EngagedMD videos when filling out the forms regarding patient comprehension.

There is also need for additional research before a practice shift in instituting MMEs. How many topics a patient can reasonably cover with an MME, what type of topics should be covered by MME vs. standard counseling, and patients’ long-term comprehension after education via an MME are all important considerations. Additionally, if providers are not adequately trained to address genetic carrier screening and opt for MME utilization in this realm, there should be a plan in place for managing positive results.

## Conclusion

In conclusion, utilization of the EngagedMD platform for the topic of genetic carrier screening resulted in both patient and provider benefits. This represents a strong starting point for the utilization of MME platforms in the field of reproductive endocrinology.

## CRediT Authorship Contribution Statement

**Annabelle Gordon:** Writing – original draft, Formal analysis. **Anthony Leonard:** Methodology, Formal analysis. **Suruchi Thakore:** Supervision, Data curation. **Kurt Peterson:** Conceptualization. **Emily Hurley:** Conceptualization. **Megan Sax:** Writing – review & editing, Methodology, Data curation.

## Declaration of Interests

A.G. has nothing to disclose. A.L. has nothing to disclose. S.T. reports support from EngagedMD (provided online portal and quizzes for patients and providers in our office free of charge for the purpose of this study) for the submitted work, Swiss Precision Diagnostics Medical Advisory Board, and Jewish Fertility Foundation Medical Advisory Board, outside the submitted work. K.P. has nothing to disclose. E.H. reports support from EngagedMD (provided online portal and quizzes for patients and providers in our office free of charge for the purpose of this study) for the submitted work. M.S. reports support from EngagedMD (provided online portal and quizzes for patients and providers in our office free of charge for the purpose of this study) for the submitted work.
