# Supplementary material for: The effect of media aids in genetic carrier screening education among patients with infertility
Source: F S Rep. 2025 Jan 9;6(1):60–6. doi: 10.1016/j.xfre.2025.01.003 (PMC11973739; doi:10.1016/j.xfre.2025.01.003)
Supplement: Supplemental Figure 3 [file mmc3.docx]

Figure 3: Patient Survey for EngagedMD Videos Arm
